# Activating an invertebrate bistable opsin with the all-trans 6.11 retinal analog

**DOI:** 10.1073/pnas.2406814121

**Published:** 2024-07-23

**Authors:** Matthew J. Rodrigues, Oliver Tejero, Jonas Mühle, Filip Pamula, Ishita Das, Ching-Ju Tsai, Akihisa Terakita, Mordechai Sheves, Gebhard F. X. Schertler

**Affiliations:** ^a^Laboratory of Biomolecular Research, Department of Biology and Chemistry, Paul Scherrer Institute, Villigen 5232, Switzerland; ^b^Department of Biology, ETH-Zurich, Zurich, Switzerland; ^c^Department of Molecular Chemistry and Materials Science, Weizmann Institute of Science, 76100 Rehovot, Israel; ^d^Department of Biology, Graduate School of Science, Osaka Metropolitan University, Osaka 558-8585, Japan; ^e^The Osaka Metropolitan University Advanced Research Institute for Natural Science and Technology, Osaka Metropolitan University, Osaka 558-8585, Japan

**Keywords:** opsins, GPCR, retinal, optogenetics, photobiology

## Abstract

Animal vision depends on opsins, a category of G protein-coupled receptor (GPCR) that achieves light sensitivity by covalent attachment to retinal. Typically binding as an inverse agonist, 11-cis retinal photoisomerizes to the all-trans isomer and activates the receptor, initiating downstream signaling cascades. Retinal bound to bistable opsins isomerizes back to the 11-cis state after absorption of a second photon, inactivating the receptor. Bistable opsins are essential for invertebrate vision and nonvisual light perception across the animal kingdom. While crystal structures are available for bistable opsins in the inactive state, it has proven difficult to form homogeneous populations of activated bistable opsins either via illumination or reconstitution with all-trans retinal. Here, we show that a nonnatural retinal analog, all-trans retinal 6.11 (ATR6.11), can be reconstituted with the invertebrate bistable opsin, Jumping Spider Rhodopsin-1 (JSR1). Biochemical activity assays demonstrate that ATR6.11 functions as a JSR1 agonist. ATR6.11 binding also enables complex formation between JSR1 and signaling partners. Our findings demonstrate the utility of retinal analogs for biophysical characterization of bistable opsins, which will deepen our understanding of light perception in animals.

Jumping Spider Rhodopsin-1 (JSR1) is a light-sensitive G protein-coupled receptor (GPCR) that the jumping spider requires for depth perception (1). It achieves light sensitivity by binding 11-cis retinal to a lysine side chain via a protonated Schiff base (PSB) (2). Retinal photoisomerization to the all-trans isomer triggers conformational rearrangements, resulting in JSR1 adopting an active conformation that catalyzes nucleotide exchange in intracellular G proteins (2, 3). JSR1 thereby transduces an optical signal to initiate cellular signaling cascades. Unlike vertebrate visual opsins, which are bleached of retinal after photoactivation, all-trans retinal bound to invertebrate and nonvisual vertebrate opsins reverts to the 11-cis state after a second photoisomerization event (4). They are therefore classified as “bistable” due to the thermal stability of the PSB in both the inactive and active states. Bistable opsins are potential optogenetic switches to control G protein signaling, and in vivo studies have used JSR1 to manipulate neuronal signaling in animals (5).

High-resolution structures of JSR1 and squid opsin elucidated differences in the retinal binding site architectures of monostable and bistable opsins (3, 6). There are currently no active state structures of bistable opsins interacting with signaling partners; it is therefore unclear how they change conformation after photoisomerization. Structural studies require a homogeneous receptor population in the active state and it has not been possible to reconstitute JSR1 with all-trans retinal. Additionally, due to the overlapped absorption maxima (λ_max_) of the inactive and active states, JSR1 illumination generates a mixture of states not easily amenable to structural characterization. The λ_max_ of bistable opsins is often similar in the active and inactive states, as shown for JSR1, melanopsin, and squid rhodopsin (2, 7, 8). Here, we use locked retinal analogs to activate JSR1 without light.

## Results

**Fig. 1.**
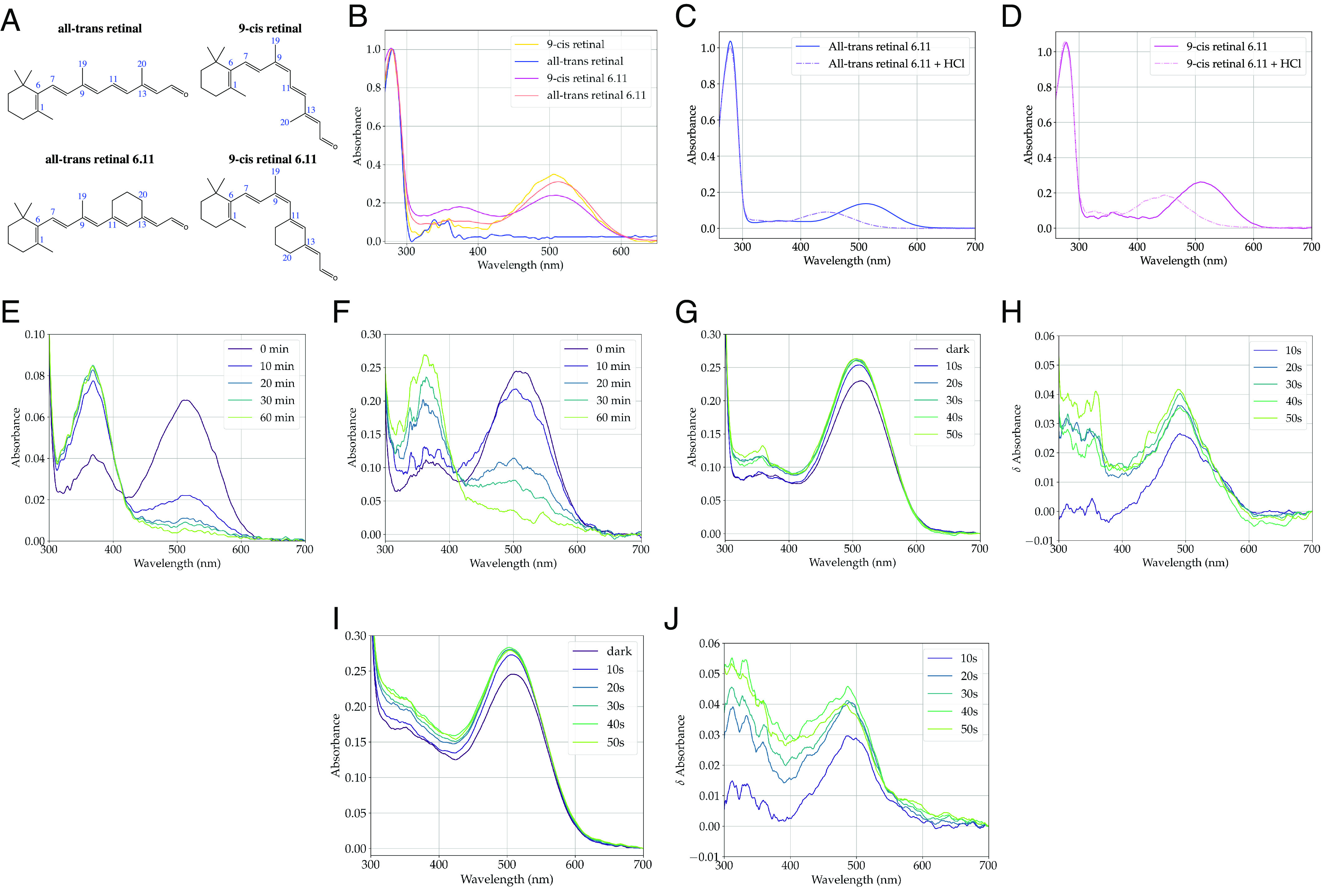
(*A*) Structures of all-trans retinal, 9-cis retinal, ATR6.11, and 9CR6.11. (*B*) UV-Vis spectra of JSR1 reconstituted with 9-cis retinal, all-trans retinal, 9CR6.11, and ATR6.11. Acid denaturation of (*C*) JSR1/ATR6.11 and (*D*) JSR1/9CR6.11. UV-Vis spectra of (*E*) JSR1/ATR6.11 and (*F*) JSR1/9CR6.11, after hydroxylamine addition. (*G* and *H*) UV-Vis and difference spectra of JSR1/ATR6.11 after illumination. (*I* and *J*) UV-Vis and difference spectra of JSR1/9CR6.11 after illumination.

JSR1 was expressed recombinantly in the apo state, addition of 9-cis retinal (Fig. 1*A*) to cell lysate enabled reconstitution with the inverse agonist, as evidenced by the 505 nm λ_max_ (Fig. 1*B*). All-trans retinal failed to reconstitute with JSR1 (Fig. 1*B*), and we therefore sought nonnatural JSR1 agonists. We reconstituted JSR1 with 9-cis retinal 6.11 (9CR6.11) and ATR6.11, which have a six-membered ring cyclized around the 11-double bond of retinal (Fig. 1*A*). JSR1 bound ATR6.11 and 9CR6.11, as demonstrated by the absorption spectra (Fig. 1*B*), with 513 nm and 509 nm λ_max_ for JSR1 bound to ATR6.11 and 9CR6.11, respectively (Fig. 1*B*).

Acid denaturation experiments confirmed covalent binding of ATR6.11 and 9CR6.11 to JSR1. The λ_max_ shifts from ≈510 nm to 445 nm (Fig. 1 *C* and *D*) for both retinal isomers after addition of HCl, as expected if the protein unfolds and the PSB becomes solvent exposed (8). The extinction coefficients of JSR1/ATR6.11 and JSR1/9CR6.11 were determined using a hydroxylamine assay, in which hydroxylamine cleaves the PSB and releases the retinal oximes (λ_max_ 365 nm). The extinction coefficient is calculated using the extinction coefficients determined for ATR6.11 and 9CR6.11, and the ratio between the 509 nm absorption decrease and the 365 nm absorption increase. We determined the extinction coefficients of JSR1/ATR6.11 and JSR1/9CR6.11 to be 49,500 M^−1^ cm^−1^ and 37,400 M^−1^ cm^−1^ at 509 nm, respectively (Fig. 1 *E* and *F*). The higher extinction coefficient of ATR6.11 compared to 9CR6.11 corresponds well to the higher extinction coefficient of JSR1 bound to all-trans retinal compared to 9-cis, 37,560 M^−1^ cm^−1^ and 32,660 M^−1^ cm^−1^, respectively (2). The A_280nm_ extinction coefficient for JSR1 based on the protein sequence is 86,330 M^−1^ cm^-1^, and the optimal A_280nm_/A_509nm_ ratios for pure JSR1 completely reconstituted with ATR6.11 and 9CR6.11 is 1.74 and 2.31, respectively. We observed A_280nm_/A_509nm_ ratios above 3.0 for both analogs, indicating that some JSR1 is not reconstituted with the analogs.

While the C11═C12 bond of the retinal analogs is constrained, other bonds remain susceptible to photoisomerization. We observe a marked absorption increase by JSR1/ATR6.11 at 491 nm within the first 10 s of illumination at 519 nm (Fig. 1 *G* and *H*). We also observe increased absorption at 488 nm within the first 10 s of illumination of JSR1/9CR6.11 (Fig. 1 *I* and *J*). While the molecular bases of these changes are unresolved, both analogs clearly remain light sensitive after binding JSR1.

**Fig. 2.**
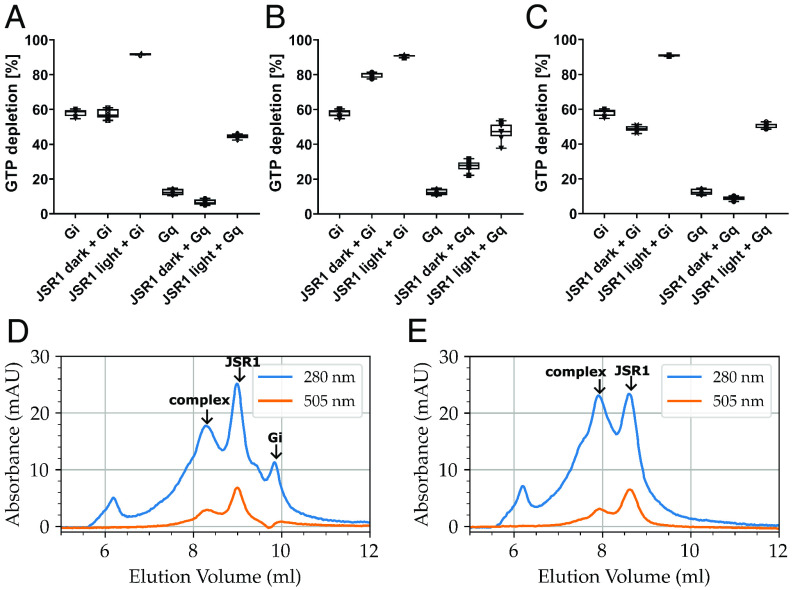
GTP depletion by human Gi and Gq heterotrimers in the presence and absence of (*A*) JSR1/9-cis retinal, (*B*) JSR1/ATR6.11, (*C*) JSR1/9CR6.11, with and without illumination. SEC profiles for JSR1/ATR6.11 incubated with (*D*) human Gi and (*E*) human Gq heterotrimers.

We then tested whether the analogs function as agonists. Activated JSR1 catalyzes exchange of GDP for GTP in the Gq heterotrimer (9) and couples to human Gi in vitro (3). A GTPase-Glo assay was used to measure GTP depletion after incubating JSR1 with either Gq or Gi heterotrimer. In the absence of JSR1, Gi has higher basal activity than Gq, with almost 60% of nucleotides depleted compared to 15% for Gq (Fig. 2). Addition of JSR1/9-cis retinal does not increase G protein nucleotide exchange (Fig. 2*A*), since 9-cis retinal stabilizes the JSR1 inactive conformation (3). Illumination of JSR1/9-cis retinal generates a mixture of JSR1 conformations, due to the overlapped absorption profiles of the 9-cis, 11-cis and all-trans isomers, with 73% adopting the all-trans conformation (2). Consistent with this, GTP depletion by Gi and Gq increased from 57% and 7% to 92% and 44%, respectively, after JSR1/9-cis retinal illumination (Fig. 2*A*).

In contrast, JSR1/ATR6.11 catalyzes nucleotide exchange without illumination, increasing GTP depletion in the presence of Gi from 58 to 80% and from 13% to 28% in the presence of Gq (Fig. 2*B*). Illuminating JSR1/ATR6.11 further increases Gi and Gq activity to 91% and 47%, respectively (Fig. 2*B*). While the ATR6.11 C11═C12 double bond is constrained in a trans configuration, illumination may induce structural changes that increase its potency as a JSR1 agonist. Dark state JSR1/9CR6.11 does not catalyze nucleotide depletion by Gi or Gq (Fig. 2*C*), similar to the effect of 9-cis retinal. JSR1/9CR6.11 illumination increases nucleotide exchange in Gi and Gq to similar levels observed for illuminated JSR1/9-cis retinal and JSR1/ATR6.11 (Fig. 2*C*). The light-induced increase in nucleotide depletion may be caused by isomerization around the 9CR6.11 C_9_═C_10_ double bond, yielding ATR6.11 chromophore. However, light induced formation of di-cis retinal analogs has also been shown to activate monostable opsins (10).

Finally, we investigated whether JSR1/ATR6.11 would form a stable complex with the Gi and Gq heterotrimers. JSR1 and G protein was incubated together in the presence of apyrase. Both samples were subjected to size exclusion chromatography and exhibited a pronounced peak at ~8.8 mL corresponding to JSR1 alone, with a relatively high ratio of absorbance at 505 nm compared to 280 nm (Fig. 2 *D* and *E*). An additional peak at 7.8 mL when JSR1/ATR6.11 is incubated with Gq (Fig. 2*E*) indicates formation of a higher molecular weight complex with a shorter retention volume. Similarly, when JSR1/ATR6.11 was incubated with Gi, a species with a shorter retention volume (8.3 mL) formed with absorbance at 280 nm and 505 nm (Fig. 2*D*). SDS-PAGE confirmed full complex formation between JSR1/ATR6.11 and the Gi and Gq heterotrimers, demonstrating that ATR6.11 induces complex formation with signaling partners (11). However, significant populations of JSR1/ATR6.11 and Gi remain unbound to each other, indicating less stable complex formation compared to Gq (Fig. 2*D*).

## Discussion

Retinal analogs have been used to investigate the photochemistry of microbial opsins (12, 13) and monostable vertebrate opsins (10, 14–16), here, we expand their use to bistable opsins. Our understanding of bistable opsins remains limited by bottlenecks in expression and reconstitution with their native chromophores. We show that reconstituting ATR6.11 with JSR1 forms an activated receptor population suitable for biophysical studies to inform our understanding of opsins. The reason for selective binding of ATR6.11 over all-trans retinal remains unclear. ATR6.11 may bind with higher affinity, or its aldehyde group may be better positioned to form a Schiff base bond with the lysine residue. We note that most bistable opsins have not evolved to bind all-trans retinal and that agonist binding would cause light-independent receptor activation. The light-dependent increase in JSR1/ATR6.11 activity suggests that some *cis* isomers of retinal 6.11 may further activate JSR1, although the nature of the photoreaction must be confirmed by HPLC analysis of retinal oximes extracted from JSR1. Whether JSR1/ATR6.11 signaling is curtailed by Arrestin, which can bind squid rhodopsin and JSR1 in a phosphorylation-independent manner, also remains an open question (17, 18). We envisage that ATR6.11 will be useful to the opsin community, as its ability to activate opsins can be easily tested using biochemical and cellular signaling assays.

## Materials and Methods

JSR1 was expressed in HEK293 GnTI- cells, solubilized in DDM detergent, and purified by 1D4 affinity chromatography. Human Gαi and Gαq subunits were expressed recombinantly in *Escherichia coli* and High Five insect cells, respectively. Spectroscopy data were processed and plotted using custom Python scripts; experimental data and processing scripts are available on Zenodo (https://zenodo.org/records/1270387) (11). Full materials and methods may be found in *SI Appendix*.

## Supplementary Material

Appendix 01 (PDF)

## Data Availability

UV-Vis spectroscopy, biochemical assay, size exclusion chromatography, gel electrophoresis data have been deposited in Zenodo (https://zenodo.org/records/12703870) (11).
